# Childhood Adversity and Adult Onset of Hypertension and Heart Disease in São Paulo, Brazil

**DOI:** 10.5888/pcd10.130193

**Published:** 2013-12-05

**Authors:** Canada Parrish, Pamela J. Surkan, Silvia S. Martins, Wagner F. Gattaz, Laura Helena Andrade, Maria Carmen Viana

**Affiliations:** Author Affiliations: Pamela J. Surkan, Johns Hopkins Bloomberg School of Public Health, Baltimore, Maryland; Silvia S. Martins, Columbia University Mailman School of Public Health, New York, New York; Wagner F. Gattaz, Laura Helena Andrade, Department/Institute of Psychiatry, University of São Paulo Medical School, São Paulo, Brazil; Maria Carmen Viana, Department of Social Medicine and Postgraduate Program in Collective Health, Federal University of Espírito Santo, Vitoria, Brazil.

## Abstract

Using data from the São Paulo Megacity Mental Health Survey and logistic regression models, we studied how childhood neglect, physical abuse, sexual abuse, and family violence were related to adult hypertension and heart disease. After adjustment for sociodemographic factors, child physical abuse was associated with hypertension and heart disease, whereas family violence was associated with hypertension. Efforts to curb child physical abuse could potentially reduce subsequent hypertension and heart disease.

## Objective

Cardiovascular disease is a leading cause of death and disability globally (1). Social and behavioral determinants starting in childhood are important for prevention (2–4). The benefits of early intervention are often observed many years later (5–7); thus, documentation of how childhood factors affect adult health is of paramount importance. The objective of this study was to examine the association of adverse childhood experiences — namely, neglect, physical abuse, sexual abuse, and family violence — with hypertension and heart disease in a sample of adults from the São Paulo, Brazil, metropolitan area.

## Methods

We used data from the São Paulo Megacity Mental Health Survey (8), a population-based cross-sectional survey of adult psychiatric morbidity and the Brazilian component of the World Health Organization (WHO) World Mental Health Surveys (8). We interviewed 5,037 participants from May 2005 through April 2007. Respondents were selected through a stratified, multistage, clustered-area probability sample of households; the survey response rate was 81.3% (8). The study was approved by the Ethical and Research Committee of University of São Paulo.

Childhood (under age 18) adversity variables included self-reported neglect, physical abuse, sexual abuse, and family violence, as defined by the WHO World Mental Health Surveys (9). For hypertension, participants were asked if they had high blood pressure readings in the previous 12 months. To assess heart disease, participants were asked if a doctor or other health professional had ever told them that they had heart disease. 

The analysis, conducted in Stata version 12 (StataCorp LP, College Station, Texas), used survey data procedures to account for weight and primary sampling units and strata information to account for the stratified, multistage, clustered-area sampling design (8). Descriptive statistics were calculated by using χ^2^ tests.

Each adversity variable was included in separate multivariable logistic regression models adjusted for sex, age (18–34, 35–49, 50–64, ≥65 y), education level (0–11, 12, 13–15, ≥16 y), and income (low, low-average, high-average, high). We included models also adjusted for onset of depression (first episode at age <18 y; first episode at age ≥18 y) by using the WHO Composite International Diagnostic Interview (8). These covariates have demonstrated significant associations with cardiovascular conditions (1,3). Finally, we fit a full model that included all 4 childhood adversity variables and the covariates. Tests were 2-sided, and the significance level was set at *P* < .05.

## Results

Of 5,037 participants, 969 (19.2%) had hypertension and 248 (4.9%) had heart disease; 546 (10.8%) reported neglect, 610 (12.1%) reported physical abuse, 26 (0.5%) reported sexual abuse, and 524 (10.4%) reported family violence (Table 1). Of the sociodemographic covariates, only increasing age was associated with higher prevalence of hypertension or heart disease. The crude odds of developing hypertension (odds ratio [OR] = 1.54; 95% confidence interval [CI], 1.21–1.95) and heart disease (OR = 1.72; 95% CI, 1.01–2.93) were greater for participants who reported childhood neglect than for those who did not. Also, the crude odds of developing hypertension (OR = 1.95; 95% CI, 1.58–2.41) and heart disease (OR = 1.96; 95% CI, 1.27–3.04) were greater for participants who reported physical abuse than for those who did not.

**Table 1:** Heart Disease and Hypertension in a Sample of Adults, by Sociodemographic Variable, Childhood Adversity, and Onset of Depression, São Paulo, Brazil, 2005–2007^a^

Characteristic	Participants, No. (%)	Hypertension		Heart Disease		
		No. (%)	Crude OR (95% CI)	No. (%)	Crude OR (95% CI)	
**Sex**						
Female	2,670 (53.0)	591 (22.2)	1 [Reference]	149 (5.6)		1 [Reference]
Male	2,367 (47.0)	378 (16.1)	0.69 (0.57–0.85)	99 (4.2)		0.77 (0.57–1.02)
**Age, y**						
18–34	2,252 (44.7)	141 (6.3)	1 [Reference]	44 (2.0)		1 [Reference]
35–49	1,604 (31.9)	302 (18.9)	3.09 (2.42–3.95)	67 (4.2)		2.41 (1.58–3.67)
50–64	772 (15.3)	323 (42.9)	9.85 (8.03–12.10)	70 (9.1)		5.48 (2.72–11.06)
≥65	410 (8.1)	197 (48.2)	12.25 (7.82–19.20)	67 (16.3)		9.76 (6.11–15.60)
**Income** b						
Low	1,132 (22.5)	240 (21.5)	1 [Reference]	43 (3.8)		1 [Reference]
Low-average	1,389 (27.5)	264 (19.1)	0.84 (0.65–1.09)	69 (5.0)		1.16 (0.80–1.66)
High-average	1,225 (24.3)	224 (18.4)	0.80 (0.62–1.04)	61 (5.0)		1.17 (0.72–1.90)
High	1,292 (25.6)	241 (18.7)	0.81 (0.60–1.11)	75 (5.8)		1.32 (0.82–2.14)
**Childhood adversity^c^**						
None	3,843 (76.3)	669 (17.5)	NA	158 (4.1)		NA
Neglect	546 (10.8)	141 (26.0)	1.54 (1.21–1.95)	41 (7.5)		1.72 (1.01–2.93)
Physical abuse	610 (12.1)	182 (30.0)	1.96 (1.58–2.41)	50 (8.3)		1.96 (1.27–3.04)
Sexual abuse	26 (0.5)	5 (17.6)	0.86 (0.29–2.58)	1 (1.8)		1.86 (0.46–7.46)
Family violence	524 (10.4)	110 (21.2)	1.14 (0.93–1.38)	26 (5.0)		1.06 (0.68–1.73)
**Depression**						
No depression	4,116 (81.7)	752 (18.4)	1 [Reference]	176 (4.3)		1 [Reference]
Early onset^d^	222 (4.4)	37 (16.8)	0.90 (0.52–1.55)	11 (4.9)		1.09 (0.55–2.16)
Adult onset	700 (13.9)	180 (25.8)	1.53 (1.22–1.91)	61 (8.8)		2.19 (1.48–3.24)

Abbreviations: OR, odds ratio; CI, confidence interval, NA, not applicable.

a Weighted counts from the survey data are rounded to the nearest whole number. The total sample size (N = 5,037) varies by missing data for each variable.

b Income levels were calculated in reference to the median income in Brazil.

c The reference group for each adversity is absence of that adversity. For example, participants reporting neglect are compared with participants who did not report neglect.

d Defined as having a first depressive episode before age 18 years.

Family violence significantly predicted hypertension (adjusted OR [AOR] = 1.48; 95% CI, 1.20–1.83) but not heart disease in models adjusted for sociodemographic factors. After adjustment for sociodemographic variables alone or for sociodemographic variables and onset of depression, neglect and sexual abuse were not significantly associated with hypertension or heart disease (Table 2). As in the models adjusted for sociodemographic varibles alone, the association between family violence and hypertension was significant in the adjusted models for depression, but it was slightly attenuated (AOR = 1.37; 95% CI, 1.10–1.71).

**Table 2:** Crude and Adjusted Odds Ratios (ORs) and 95% Confidence Intervals (CIs) of Single and Multiple Childhood Adversities Predicting Hypertension and Heart Disease, São Paulo, Brazil, 2005–2007

Adversity	Model 1: Crude OR (95% CI)	Model 2: Adjusted OR (95% CI)^a^	Model 3: Adjusted OR (95% CI)^b^	Model 4: Adjusted OR (95% CI)^c^
**Hypertension**				
Neglect	1.54 (1.21–1.95)	1.15 (0.84–1.59)	1.11 (0.80–1.53)	0.96 (0.69–1.33)
Physical abuse	1.95 (1.58–2.41)	1.76 (1.38–2.25)	1.64 (1.30–2.07)	1.59 (1.22–2.07)
Sexual abuse	0.86 (0.29–2.58)	1.08 (0.36–3.27)	0.90 (0.32–2.53)	0.74 (0.27–2.07)
Family violence	1.14 (0.93–1.38)	1.48 (1.20–1.83)	1.37 (1.10–1.71)	1.18 (0.94–1.49)
**Heart disease**				
Neglect	1.72 (1.01–2.93)	1.43 (0.81–2.50)	1.31 (0.74–2.31)	1.21 (0.67–2.16)
Physical abuse	1.96 (1.27–3.04)	1.74 (1.06–2.85)	1.46 (0.88–2.43)	1.40 (0.85–2.32)
Sexual abuse	1.86 (0.46–7.46)	2.60 (0.67–10.07)	1.80 (0.48–6.83)	1.59 (0.43–5.93)
Family violence	1.06 (0.65–1.73)	1.34 (0.79–2.27)	1.16 (0.69–1.96)	0.96 (0.57–1.63)

a Model 2 adjusted for sex (male, female), age (18–34, 35–49, 50–64, ≥65 y), education (0–11, 12, 13–15, ≥16 y) and income (low, low-average, high-average, high).

b Model 3 adjusted for the same variables as Models 2 plus onset of depression (no depression, early onset [first depressive episode before age 18 years], adult onset [first depressive episode at age 18 years or older]).

c Model 4 adjusted for the same variables as Model 3 plus all childhood adversity variables simultaneously.

In the model adjusted for sociodemographic variables, participants reporting childhood physical abuse were significantly more likely to have adult hypertension (AOR = 1.76; 95% CI, 1.38–2.25) and heart disease (AOR = 1.74; 95% CI, 1.06–2.85). When we controlled for depression, the likelihood of hypertension was attenuated (AOR = 1.64; 95% CI, 1.30–2.07), and the association with heart disease was no longer significant (Table 2).

In the full model, which contained all 4 childhood adversities and all covariates, only physical abuse was significantly associated with hypertension (AOR = 1.59; 95% CI, 1.22–2.07), and the previously significant association between family violence and hypertension disappeared (Table 2).

## Discussion

Participants reporting childhood physical abuse were more likely to develop hypertension and heart disease than who did not report such abuse. Additionally, family violence was associated with a greater likelihood of adult hypertension.

In the model that included all 4 adversities simultaneously, only physical abuse was significantly associated with hypertension. Thus, of the 4 adversities and in the context of our study, physical abuse may be the more salient predictor of hypertension and heart disease, because childhood adversities often occur simultaneously (2,9,10). A plausible mechanism is that stress in early life triggers greater sensitivity of the hypothalamic–pituitary–adrenal axis, which then results in physiological consequences (1,11). Also, maltreatment early in life can alter psychological development (12), possibly leading later in life to poor health choices (eg, unhealthful diet, tobacco use) that contribute to the development of hypertension (2,12).

To our knowledge, apart from the WHO pooled analyses (2,10), no studies exclusively from low- or middle-income countries have reported on the association of childhood adversities with cardiovascular health. For heart disease, our study demonstrated an effect size for physical abuse similar to that found in the literature, but we found a larger effect for family violence and a smaller effect for sexual abuse (2,3,10). The effect of these adversities on adult health appears to vary by location (2), which may allude to an important contextual influence (2,9).

One limitation of our study is that the variables were assessed via self-report and were not verified by medical records. The use of self-report may result in underreporting of these outcomes, particularly in non-Western countries (10). Also, because few survey participants reported sexual abuse and heart disease was fairly uncommon, our study may have been underpowered. Therefore, it may be difficult to draw strong conclusions from our data.

Our findings highlight that support for early childhood interventions are critical. When funds for early interventions are limited, our findings may help inform more targeted interventions (3,5). Because some childhood adversity effects appear stronger than others, further study is warranted on the mechanisms driving the effects.

## References

[1] Joynt KE , Whellan DJ , O’Connor CM . Depression and cardiovascular disease: mechanisms of interaction. Biol Psychiatry 2003;54(3):248–61. 10.1016/S0006-3223(03)00568-7 12893101

[2] Scott KM , Von Korff M , Angermeyer MC , Benjet C , Bruffaerts R , de Girolamo G , Association of childhood adversities and early-onset mental disorders with adult-onset chronic physical conditions. Arch Gen Psychiatry 2011;68(8):838–44. 10.1001/archgenpsychiatry.2011.77 21810647 PMC3402030

[3] Batten SV , Aslan M , Maciejewski PK , Mazure CM . Childhood maltreatment as a risk factor for adult cardiovascular disease and depression. J Clin Psychiatry 2004;65(2):249–54. 10.4088/JCP.v65n0217 15003081

[4] Fuller-Thomson E , Brennenstuhl S , Frank J . The association between childhood physical abuse and heart disease in adulthood: findings from a representative community sample. Child Abuse Negl 2010;34(9):689–98. 10.1016/j.chiabu.2010.02.005 20663556

[5] Benjet C , Borges G , Medina-Mora ME . Chronic childhood adversity and onset of psychopathology during three life stages: childhood, adolescence and adulthood. J Psychiatr Res 2010;44(11):732–40. 10.1016/j.jpsychires.2010.01.004 20144464

[6] Karoly LA , Greenwood PW , Everingham SS , Houbé J , Kilburn MR . Investing in our children: what we know and don’t know about the costs and benefits of early childhood interventions. Santa Monica (CA): Rand Corporation; 1998.

[7] Cromer KR , Sachs‐Ericsson N . The association between childhood abuse, PTSD, and the occurrence of adult health problems: moderation via current life stress. J Trauma Stress 2006;19(6):967–71. 10.1002/jts.20168 17195985

[8] Viana MC , Teixeira MG , Beraldi F , Bassani IS , Andrade LH . São Paulo Megacity Mental Health Survey — a population-based epidemiological study of psychiatric morbidity in the São Paulo metropolitan area: aims, design and field implementation. Rev Bras Psiquiatr 2009;31(4):375–86. 10.1590/S1516-44462009000400016 20098829

[9] Kessler RC , McLaughlin KA , Green JG , Gruber MJ , Sampson NA , Zaslavsky AM , Childhood adversities and adult psychopathology in the WHO World Mental Health Surveys. Br J Psychiatry 2010;197(5):378–85. 10.1192/bjp.bp.110.080499 21037215 PMC2966503

[10] Stein DJ , Scott K , Abad JMH , Aguilar-Gaxiola S , Alonso J , Angermeyer M , Early childhood adversity and later hypertension: data from the World Mental Health Survey. Ann Clin Psychiatry 2010;22(1):19–28. 20196979 PMC3486699

[11] Rosmond R , Björntorp P . The hypothalamic–pituitary–adrenal axis activity as a predictor of cardiovascular disease, type 2 diabetes and stroke. J Intern Med 2000;247(2):188–97. 10.1046/j.1365-2796.2000.00603.x 10692081

[12] Norman RE , Byambaa M , De R , Butchart A , Scott J , Vos T . The long-term health consequences of child physical abuse, emotional abuse, and neglect: a systematic review and meta-analysis. PLoS Med 2012;9(11):e1001349. 10.1371/journal.pmed.1001349 23209385 PMC3507962

